# Pattern activation/recognition theory of mind

**DOI:** 10.3389/fncom.2015.00090

**Published:** 2015-07-15

**Authors:** Bertrand du Castel

**Affiliations:** Schlumberger ResearchHouston, TX, USA

**Keywords:** recurrent, neural, stochastic, autapse, self-description, metaphor, grammar, hylomorphism

## Abstract

In his 2012 book *How to Create a Mind*, Ray Kurzweil defines a “Pattern Recognition Theory of Mind” that states that the brain uses millions of pattern recognizers, plus modules to check, organize, and augment them. In this article, I further the theory to go beyond pattern recognition and include also pattern activation, thus encompassing both sensory and motor functions. In addition, I treat checking, organizing, and augmentation as patterns of patterns instead of separate modules, therefore handling them the same as patterns in general. Henceforth I put forward a unified theory I call “Pattern Activation/Recognition Theory of Mind.” While the original theory was based on hierarchical hidden Markov models, this evolution is based on their precursor: stochastic grammars. I demonstrate that a class of self-describing stochastic grammars allows for unifying pattern activation, recognition, organization, consistency checking, metaphor, and learning, into a single theory that expresses patterns throughout. I have implemented the model as a probabilistic programming language specialized in activation/recognition grammatical and neural operations. I use this prototype to compute and present diagrams for each stochastic grammar and corresponding neural circuit. I then discuss the theory as it relates to artificial network developments, common coding, neural reuse, and unity of mind, concluding by proposing potential paths to validation.

## Introduction

In his book *How to Create a Mind* (Kurzweil, 2012), Ray Kurzweil proposes to model the brain with a unified processing paradigm (pp. 5, 7, 23) that consists in a hierarchy of self-organizing pattern routines (pp. 29, 172) “…*constantly predicting the future and hypothesizing what we will experience*” (pp. 31) and “…*involved in our ability to recognize objects and situations*” (pp. 32). Furthermore, Kurzweil proposes that the model be implemented with hierarchical hidden Markov models (HHMMs, p. 144) whose parameters are learned via genetic algorithms (p. 146).

Importantly, Kurzweil calls his model “Pattern Recognition Theory of Mind.” While I fully concur to the hypothesis of a unified processing paradigm involving patterns, I suggest in this article that the model Kurzweil presents in his book does not fully achieve his goal and that it can be more ambitious. First, I note that Kurzweil adds to the central pattern recognition processing two modules: a “critical thinking module” (p. 175) that checks for consistency of patterns, and an “open question module” (p. 175) that uses metaphors to create new patterns. These two modules invoke additional, separate apparatus to that of his pattern recognition modules. Second, Kurzweil proposes that self-organization of pattern recognition modules is achieved via linear programming (p. 174), which is yet another apparatus.

I suggest that while functions of consistency checking, metaphorical creation, and self-organization are indeed needed, their treatment should not require extra mechanisms. The theory should handle them just as any other pattern, since they are actually patterns of patterns, an assertion that I will formally substantiate further on. Moreover, I consider that limiting pattern processing to recognition is limiting the scope of a unified processing paradigm. Indeed, patterns of activation are not different from patterns of recognition; for example, drawing a circle or recognizing a circle both involve the same pattern (circle), and so the same unified pattern mechanism should apply in both cases, with the difference being determined only by the type of action (Mumford and Desolneux, 2010). In fact, Kurzweil does mention that HHMMs can be used in both activation and recognition (p. 143); but he did not include that in his model, nor did he consider them being used *at the same time* in activation and recognition, a key element of my analysis going forward.

In this article, I therefore advance an evolution of the model, extending it to both activation and recognition. I call it “Pattern Activation/Recognition Theory of Mind.” It is based on stochastic grammars, i.e., the superset model from which HHMMs were originally derived (Fine et al., 1998). Stochastic grammars differ from HHMMs in that they are fully recursive, or, said otherwise, fully recurrent. I will demonstrate that they are capable of self-description, allowing them to handle both patterns and patterns of patterns. Consequently, they can account for pattern consistency, since consistency is a pattern of patterns; similarly, stochastic grammars can use metaphors to create new patterns in a process which also involves patterns of patterns. Since the self-describing stochastic grammars I present can both activate and recognize patterns, they meld in a single model the motor and sensory experiences of the brain.

More generally, I will show that in addition to providing a unified processing paradigm, self-describing stochastic grammars capable of both activation and recognition have a natural mapping to neuronal circuits. Recursion (recurrence), which enables self-description, is a property of neural circuits, an example being a neuron whose axon feeds back to the neuron itself via an autapse (van der Loos and Glaser, 1972). In addition, stochastic grammars probabilities express the stochastic nature of synapses with excitation (more likely to trigger; probability above .5) and inhibition (less likely to trigger; probability below .5). Furthermore, activation is what an axon does, while recognition is what a dendrite does. Self-description allows reading, modifying, and creating neuronal circuitry, modeled by stochastic grammars both activating and recognizing other stochastic grammars. This is equivalent to saying that patterns can both activate and recognize other patterns, thus providing a general mechanism to create, modify, and exercise patterns and patterns of patterns for both activation and recognition.

In order to make the correspondence between stochastic grammars and neural circuits both clear and explicit, I present each grammar of the text together with an associated neural diagram. Neural properties to watch for are those that map stochastic grammars properties: hierarchy, recurrence, directionality, probabilities, and, most importantly for this presentation, learning afforded by self-description. Diagrams are produced by running a prototype implementation of the model in the form of a probabilistic programming language belonging to the lineage of PRISM (Sato and Kameya, 1997) and Church (Goodman et al., 2008), but dedicated to the self-describing activation/recognition grammatical and neural operations described in this article.

In summary, I present here a model that augments and completes Kurzweil's software ambition and has a natural interpretation in terms of the brain's hardware. Subsequently, I discuss the theory as it relates to artificial network developments, common coding, neural reuse, and unity of mind, and I propose potential paths to validation. Finally, Kurzweil places evolution at the center of his learning model, with genetic algorithms. I prefer here to use instead swarming as a learning mechanism, as evolution is a phylogenetic property (which happens over time), while swarming is an ontogenetic property (which happens in real time). Swarming is readily achieved with neuronal circuits in lower and higher animals, and therefore readily asserted in neural circuits. With this considered, I can now describe the Pattern Activation/Recognition Theory of Mind.

## Materials and methods

I first introduce activation/recognition grammars, a class of probabilistic context-free grammars that can function both separately and simultaneously in activation and recognition. I show that activation/recognition grammars have the power needed for biologically-inspired computations, that is, the power of Turing machines, while providing an additional level of expressiveness and theory that maps neural circuitry. I demonstrate with an example from vision how a probabilistic version of these grammars can learn colors through reinforcement by expressing a swarm of lower grammars. I then show with metaphor and composition that this mechanism generalizes up to a self-describing grammar that provides the root of a hierarchical and recursive unified model of pattern activation and recognition in neural circuits.

### Activation/recognition

I now introduce grammars that can simultaneously activate and recognize patterns.

A grammar executes a set of rules until rules no longer apply (Chomsky, 1957). Grammars herein are probabilistic (a.k.a. stochastic) context-free grammars without null symbol (Booth, 1969; Chi, 1999). They are coded in a minimal subset of Wirth's syntax notation (Wirth, 1977), augmented with probabilities. These grammars are not traditional, because they are not limited to functioning solely either in activation or recognition; instead, they can function *both separately and simultaneously* in activation and recognition, which I will show is a small but critically consequential specification in regard to biologically-inspired interpretation of grammar theory and practice.

Grammar “**Draw** = *DrawSquare DrawCircle DrawTriangle*.” produces a square, a circle, and a triangle. Conversely, when presented with a square, a circle, and a triangle, grammar “**Spot** = *SpotSquare SpotCircle SpotTriangle*.” recognizes them. Production and recognition can mix, as with grammar “**Mix** = *DrawSquare SpotCircle DrawTriangle*.” This grammar recognizes a circle, and produces a square and a triangle.

**Figure 1:**
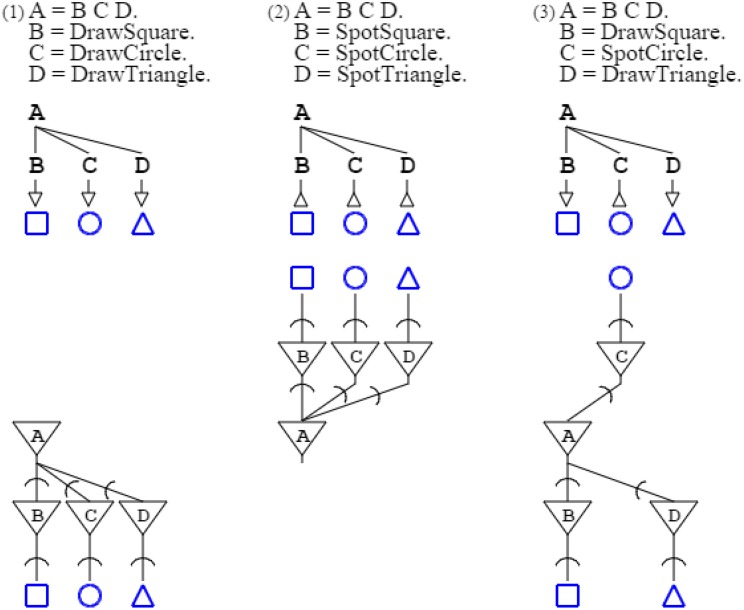
**Three grammars are shown in the top row, while the second row shows a graphical form of the grammars, and the third row presents their associated neural circuits**. Arrows in grammatical descriptions indicate when a grammar is activating (down arrow), and when it is recognizing (up arrow). In neural descriptions, an inverted triangle denotes a soma, while a segment cut by an arc denotes an axonal extension and a synapse, further connecting to a dendrite.

Formally, the three grammars above are unified in a single paradigm of production and recognition; this is captured by a common rule “**A** = **B C D**.” that expresses, with arbitrary symbols (de Saussure, 1916), the abstract pattern constituted by any three entities. This pattern is then specialized with rules that terminate abstract symbols with activation and recognition functions. The first grammar above is thus formally written “**A** = **B C D**. **B** = *DrawSquare*. **C** = *DrawCircle*. **D** = *DrawTriangle*.” while the second one is written “**A** = **B C D**. **B** = *SpotSquare*. **C** = *SpotCircle*. **D** = *SpotTriangle*.” and the third one “**A** = **B C D**. **B** = *DrawSquare*. **C** = *SpotCircle*. **D** = *DrawTriangle*.” (Figure 1).

**Figure 2:**
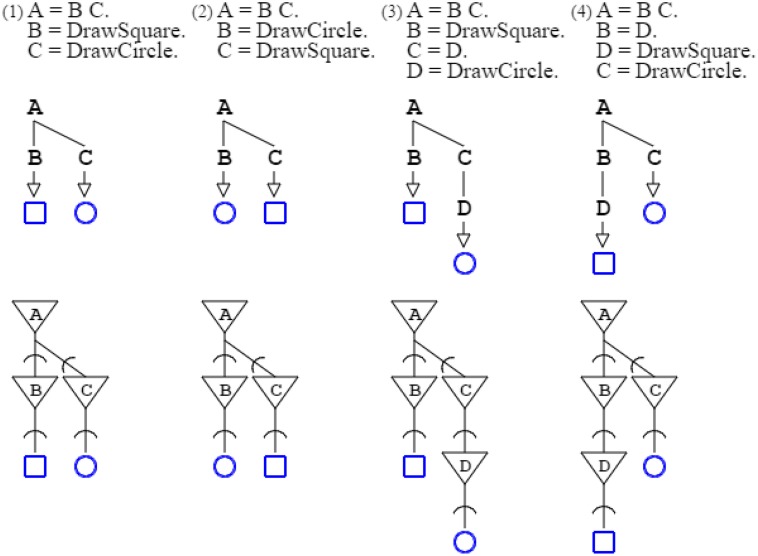
**Four grammars are shown with their neural counterpart**. The two first grammars are equivalent, as their ordering is not set by rule sequencing but rather by the number of rules applied; the production of the square requires just two rule applications, the same as for the circle. The third grammar is not equivalent to the two preceding ones, as three rule applications are required for the circle, and two for the square. The fourth grammar is different from the three preceding grammars, as the square requires three rule applications, and is therefore ordered after the circle, which takes two.

In activation/recognition grammar rules, sequencing does not convey order, except that recognition comes ahead of production. Grammars “**A** = **B C**. **B** = *DrawSquare*. **C** = *DrawCircle*.” and “**A** = **B C**. **B** = *DrawCircle*. **C** = *DrawSquare*.” are equivalent. They both produce a square and a circle, in any order. The number of rules involved in a derivation conveys order, whether for production or recognition. In the two previous examples, the number of rules needed to produce a square and a circle is the same for both grammars: two rules are involved, so the grammars are equivalent. But the grammar “**A** = **B C**. **B** = *DrawSquare*. **C** = **D**. **D** = *DrawCircle*.” is not equivalent to preceding grammars, as drawing a square requires the application of two rules, whereas drawing a circle requires the application of three rules; consequently, the square is ordered ahead of the circle (Figure 2).

**Figure 3:**
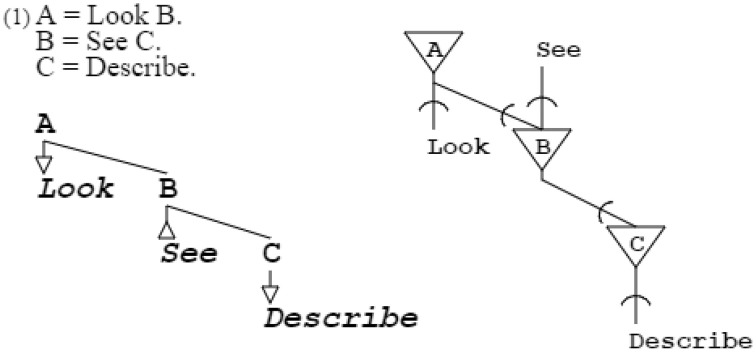
**This grammar and its neural equivalent demonstrate variety of purpose; here, actions are described instead of geometrical figures**. A, B, and C are non-terminals, while *Look, See*, and *Describe* are terminals. *Look* and *Describe* are in production, *See* is in recognition. The particular grammatical and neuronal pattern of this example will be repeatedly found further down in self-describing grammars and their neural equivalents.

Further varying the rules, infinity of situations can be conveyed. For example, grammar “**A** = *Look* **B**. **B** = *See* **C**. **C** = *Describe*.” expresses looking for an object and, on seeing it, describing it (Stock and Stock, 2004). Abstract symbols such as **A**, **B**, and **C** that expand to other symbols, are called non-terminal and have no other role than structural. Symbols such as *DrawSquare, SpotCircle, Look*, and *See* that do not expand further, are called terminal and have a functional role; as explained later, they can also be placeholders for further grammatical expansion. Terminals can be actuators or sensors; *DrawSquare* and *Look* are actuators, as they produce output, while *SpotCircle* and *See* are sensors, as they recognize input (Figure 3).

Taken as a whole, the above account of grammars differs from tradition (du Castel, 1978) only in that it melds activation and recognition, or, otherwise stated, actuation, and sensing, or action/perception (Clark, 2013). Grammars have long been known to work alternatively in production (“performance”) and recognition (“competence”), but their working *simultaneously* in both modes is new; and with this small addition, activation/recognition grammars find a new role in biologically-inspired computation.

### Expressivity

I now add probabilities to activation/recognition grammars, and I show that these grammars have the same power as Turing machines, while presenting additional modeling capabilities.

**Figure 4:**
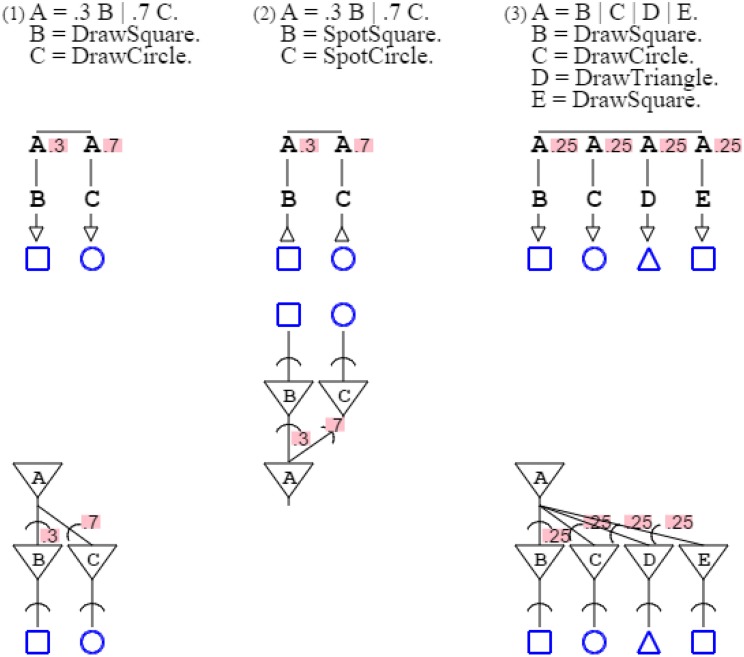
**Three grammars and their neural equivalent are presented, with probabilities**. In grammars, alternate paths are marked by a horizontal bar, with corresponding probabilities indicated for each path. In neural circuits, probabilities are associated with synapses.

Rules can have alternates, marked by the symbol “| ” (or) and weighted with probabilities. Grammar “**A** = .3 **B** | .7 **C**. **B** = *DrawSquare*. C = *DrawCircle*.” prints a square with probability .3 and a circle with probability .7, such that the mean of the grammar's distribution is 30% squares and 70% circles. Grammar “**A** = .3 **B** | .7 **C**. **B** = *SpotSquare*. **C** = *SpotCircle*.” recognizes a square with probability .3, and a circle with probability .7, such that the grammar's expectation is 30% squares and 70% circles. Note that grammar probabilities sum up to 1 for convenience of presentation, but this is not necessary; weights can substitute to probabilities without affecting the descriptive value of a grammar (Smith and Johnson, 2007). For style, unspecified probabilities are read as equipartition, such that grammar “**A** = **B** | **C** | **D** | **E**.” is actually “**A** = .25 **B** | .25 **C** | .25 **D** | .25 **E**.” (Figure 4).

**Figure 5:**
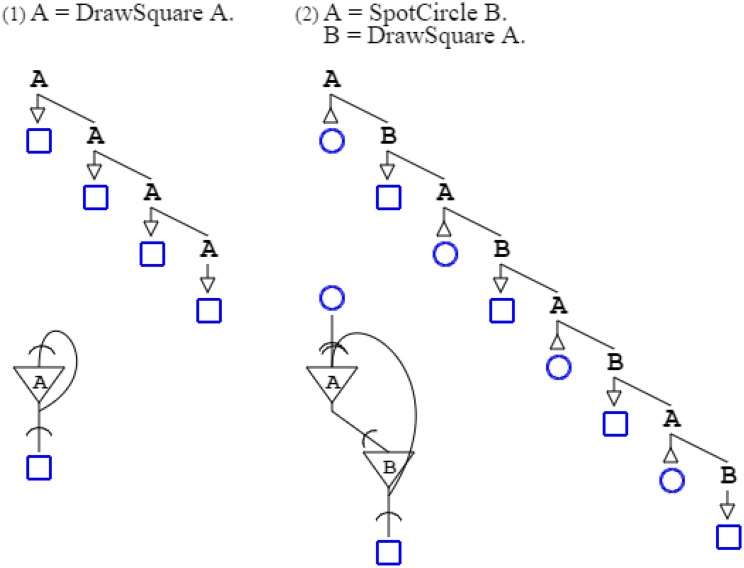
**Two recursive (recurrent) grammars are shown with their neural circuits**. In the grammatical schema, the recursion is only shown for three levels for reason of presentation (in further figures it is typically cut to one or two for the sake of clarity). The neural diagram does not suffer from the same limitation of presentation, so it is faithful to the original grammatical formulation. The recurring synapse of the first circuit connects the soma onto itself, so it categorizes as autapse. The second neural circuit has two synapses terminating on the same soma, one being recurrent. The recurrent synapse of the second circuit is categorized as regular, as it connects one soma to a different one.

Completing this enumeration of fundamental properties of activation/recognition grammars, non-terminal symbols can be used in feedback loops (Bellman, 1986; Buzsáki, 2006; Joshi et al., 2007). Grammar “**A** = *DrawSquare* **A**.” repeats producing squares to infinity, while grammar “**A** = *SpotCircle* **B**. **B** = *DrawSquare* **A**.” only repeats as long as circles are recognized, drawing as many squares as there are circles (Figure 5).

**Figure 6:**
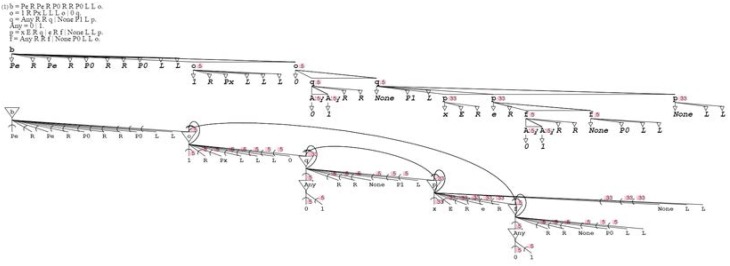
**Here is the Turing grammar with its neural circuit**. Note that the grammar has actually been simplified for the sake of presentation, as ordering should be introduced following the precepts previously enunciated regarding the number of rules executed.

Equipped with alternates and recursion, activation/recognition grammars can express Turing machines (Turing, 1936). For proof, I consider the seminal Turing example, which is the generation of the infinite sequence 001011011101111…The following activation/recognition grammar transliterates Turing's specification using its exact original notation: “**b** = *Pe R Pe R P0 R R P0 L L* **o**. **o** = *1 R Px L L L* **o** | *0* **q**. **q** = **Any** *R R* **q** | *None P1 L* **p**. **Any** = *0* | *1*. **p** = *x E R* **q** | *e R* **f** | *None L L* **p**. **f** = **Any** *R R* **f** | *None P0 L L* **o**.” In the terminology of activation/recognition grammars, terminals *Pe* (Print e), *R* (Move right), *P0* (Print 0), *L* (Move left), *Px* (Print x), *P1* (Print 1), and E (Erase), are actuators, while terminals *1* (Read 1), *0* (Read 0), *None* (Read None; meaning blank square), *x* (Read x), and *e* (Read e), are sensors. Therefore, mixed production and recognition overcomes computational limitations of traditional context-free grammars (Chomsky and Schützenberger, 1963), allowing them to perform most biologically-inspired computations, if not all (Penrose, 1989; Siegelmann, 1995) (Figure 6).

**Figure 7:**
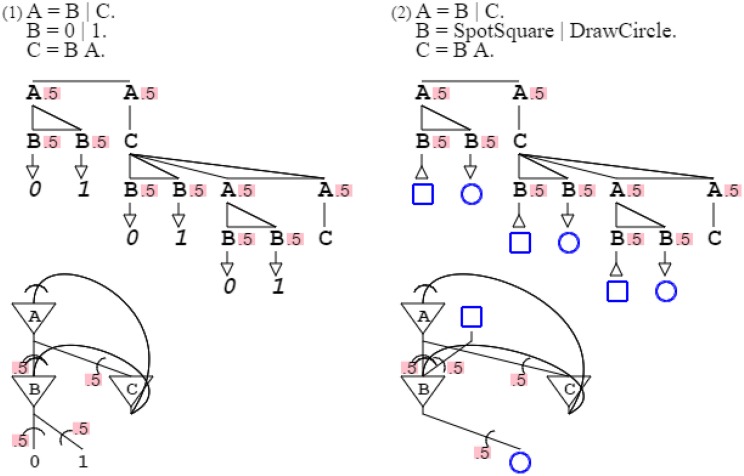
**Varying terminals**. The first grammar and its neural circuit produce binary digit sequences. The second grammar is the same as the first one except for its terminals. Instead of producing digits *0* and *1*, it recognizes squares and produces circles. It does that following the same pattern, where production of *0* is replaced by recognition of a square, and production of *1* by production of a circle. As will be discussed further down, this constitutes a metaphor, applying a set pattern (expressed by the non-terminal part of the grammar) from one domain (digits) to another (geometrical figures) by varying the terminals.

Turing's infinite sequence is actually an enumeration of the natural number set, perhaps not a biological object. However, recognition of numbers is certainly a common and early human activity (Libertus et al., 2011). Grammar “**A** = **B** | **C**. **B** = *0* **|** *1*. **C** = **B A**.” expresses any digit sequence; the first rule differentiates finishing a sequence and pursuing one, the second rule produces digits, and the third rule adds to sequences. Following the same pattern, but with different terminals, grammar “**A** = **B** | **C**. **B** = *DrawSquare* **|** *DrawCircle*. **C** = **B A**.” produces a number of squares and circles in sequences mapping the production of digits, grammar “**A** = **B** | **C**. **B** = *SpotSquare* **|** *SpotCircle*. **C** = **B A**.” recognizes such a sequence, and grammar “**A** = **B** | **C**. **B** = *SpotSquare* **|** *DrawCircle*. **C** = **B A**.” mixes identifying squares and producing circles. The non-terminal grammar part common to digits and geometrical figures expresses a metaphor from one domain (digits) to another (figures), a subject I will come back to further on (Figure 7).

I have showed that activation/recognition grammars have the same power as Turing machines, to formally demonstrate that these grammars are universal. However, they are more than Turing machines. They constitute a higher-level model that expresses in particular hierarchical and recursive (recurrent) concepts that are not explicit in Turing machines. It is this extra modeling capability that I use to build the theoretical apparatus that on one hand meets the extra requirements I have put on Kurtzweil's original theory, and on the other finds a natural expression in neural circuits.

Now, while it is yet possible to suggest that they are natural models of biologically-inspired processes, a plausible origin has to be found for activation/recognition grammars.

### Learning

I now show that probabilistic activation/recognition grammars can learn with swarms, using color learning as an example.

**Figure 8:**
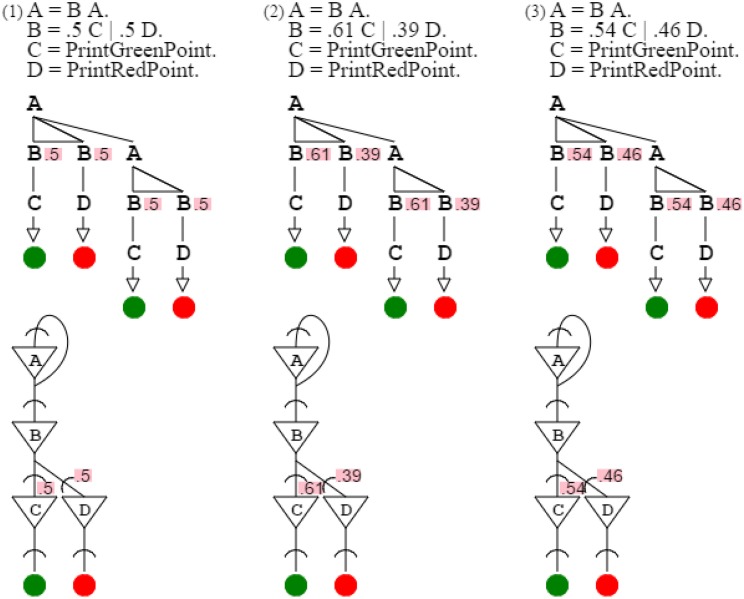
**Yellow, orange, and gold grammars are shown with their neural circuits**. The only difference between these three grammars is probabilities, which express the mixture of green and red constitutive of each color.

The use of probabilities allows producing and recognizing patterns expressing gradients. Assuming base colors red and green, grammars can produce from them composite colors, such as yellow grammar “**A** = **B A**. **B** = .5 **C** | .5 **D**. **C** = *PrintGreenPoint*. **D** = *PrintRedPoint*.” and orange grammar “**A** = **B A**. **B** = .61 **C** | .39 **D**. **C** = *PrintGreenPoint*. **D** = *PrintRedPoint*.” or gold grammar “**A** = **B A**. **B** = .54 **C** | .46 **D**. **C** = *PrintGreenPoint*. **D** = *PrintRedPoint*.” Replacing actuators by sensors, “**C** = *ReadGreenPoint*. **D** = *ReadRedPoint*.” lets grammars recognize, instead of produce, corresponding colors with variations respecting probability distributions. Yellow, orange, and gold grammars are very similar, since they differ only by their gradient probabilities (Figure 8).

The purpose of this section is to study reinforcement learning with grammar swarms. Swarms are well-understood (Kennedy and Eberhart, 1995), and grammar swarms have been studied in production (von Mammen and Jacob, 2009). Here I will consider one aspect of swarm learning, albeit a critical one, in recognition. I will consider the capability of a swarm to select for the orange color. The problem is equivalent to that of a swarm exploring for food (Dasgupta et al., 2010); instead of sampling a geometrical space, the swarm samples the green-red spectrum. The swarm is made of color grammars with different probabilities. The swarm is presented with varied colors; when a color is presented, the grammar of the swarm closest to that color recognizes it. If the recognized color is close to orange, the described grammar produces orange in return. This is the essence of swarm learning, as the swarm can then be modified to concentrate more and more on the exact orange color, a process that I do not describe here but that can be performed by the methods presented in the next section.

Color grammars presented above differ only by their probabilities. Grammars can themselves be represented by grammars (Harris, 1968). Therefore, patterns (such as individual color grammars, with set probabilities) can be represented by other patterns (such as general color grammars, with varying probabilities). My demonstration consists in showing that a grammar can describe another one in a way that expresses the swarm capability sought.

**Figure 9:**
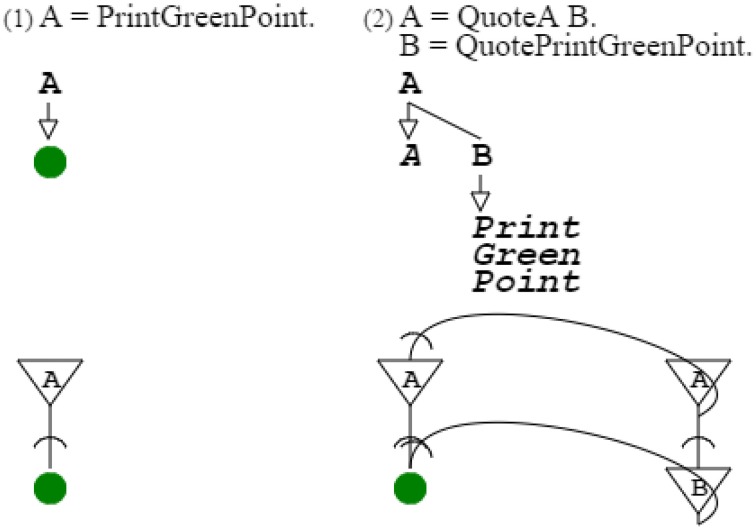
**Simple description**. The first grammar is the very simple grammar. The second grammar describes the first grammar, using the quote operator, to point to appropriate nodes of the grammar described. The second neural circuit combines the neural circuits of the describing and described grammars. The grammatical function of the quote operator is fulfilled by the projection of the neural circuit of the describing grammar to the neural circuit of the described grammar.

For this, I first need to introduce a simple case of a grammar describing another one. The most simple grammar I can consider is “**A** = *PrintGreenPoint*.” The key elements of the grammar are **A** and *PrintGreenPoint*. The equal sign (“ = ”) and dot (“.”) are markers that are necessary in the linear form of grammar writing, but that do not need to be reproduced in a grammar-describing grammar, because their function is accomplished by the structure of that grammar (cf. Methods Summary further below). Thus, grammar “**A** = *PrintGreenPoint*.” is fully described by grammar “**A** = *QuoteA* **B**. **B** = *QuotePrintGreenPoint*.” This describing grammar uses the *quote* operator. This operator allows specifying a symbol that is not otherwise active, which is necessary to avoid confusion between the non-terminal node **A** of the describing grammar and the terminal node *A* referring to the described grammar. This operator, recognized as such by Harris (Harris, 1968), is that of Gödel's proposition-describing propositions (Gödel, 1931/1986); it was also identified, independently, by McCarthy (1960) and Wirth (1977). Without this operator, the describing grammar would go into inappropriate recursion. Instead it describes the simple grammar as desired; expressing that the recursion domain of the described grammar is different from that of the describing grammar (Figure 9).

**Figure 10:**
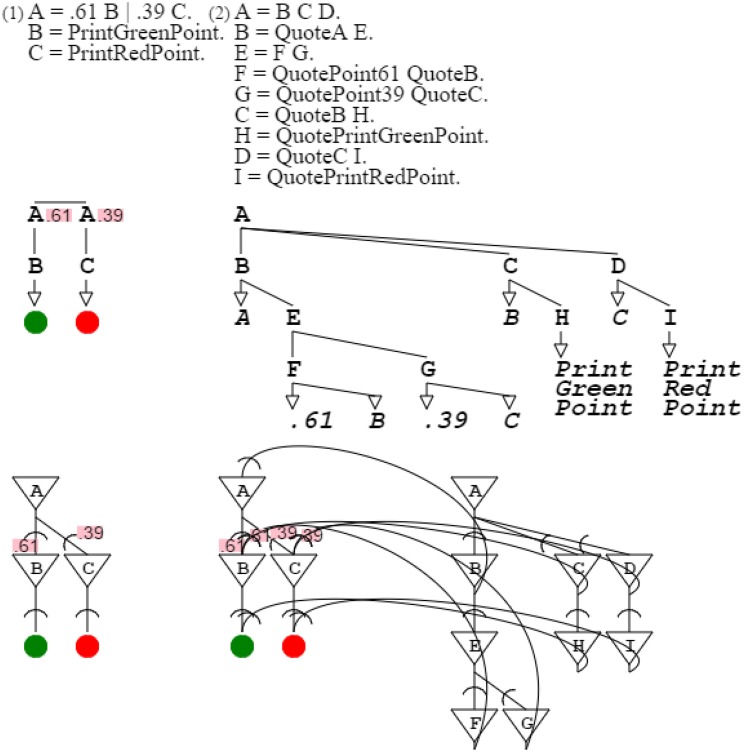
**Probabilistic description**. The first grammar is a subset of the orange grammar. The second grammar describes the first grammar. The second neural circuit combines the neural circuits of the describing and described grammars. Probabilities quoted in the describing grammar project to the described grammar, just as probabilities referred by the describing circuit project to the circuit described.

I now consider the slightly more complex grammar “**A** = .61 **B** | .39 **C**. **B** = *PrintGreenPoint*. **C** = *PrintRedPoint*.” It is a subset of the orange grammar, with probabilities expressing the proper mix of green and red that produces orange. Grammar “**A** = **B C D**. **B** = *QuoteA* **E**. **E** = **F G**. **F** = *QuotePoint61 QuoteB*. **G** = *QuotePoint39 QuoteC*. **C** = *QuoteB* **H**. **H** = *QuotePrintGreenPoint*. **D** = *QuoteC* **I**. **I** = *QuotePrintRedPoint*.” describes this orange grammar subset, including probabilities (Figure 10).

**Figure 11:**
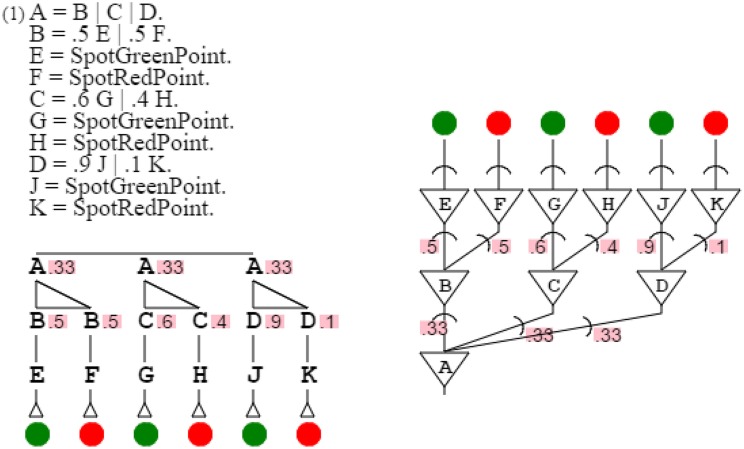
**A grammar swarm**. This is a swarm of three color grammars, side by side with its neural equivalent.

The more complex orange grammar is just an expansion of the simpler one to multiple points. For the sake of simplicity, I will therefore consider the simplified orange grammar for my demonstration. Varying the probabilities of the grammar, it is possible to produce any color grammar of the green-red spectrum. For example, it is possible to produce a grammar that recognizes colors with probabilities .5 and .5, as easily as one recognizing probabilities .6 and .4, or one recognizing .9 and .1. It is then possible to produce these three grammars at once with grammar “**A** = **B** | **C** | **D**. **B** = .5 **E** | .5 **F**. **E** = *SpotGreenPoint*. **F** = *SpotRedPoint*. **C** = .6 **G** | .4 **H**. **G** = *SpotGreenPoint*. **H** = *SpotRedPoint*. **D** = .9 **J** | .1 **K**. **J** = *SpotGreenPoint*. **K** = *SpotRedPoint*.” This assembly of grammars all similar but for one element is called a grammar swarm (von Mammen and Jacob, 2009). When presented with a color, this swarm of three grammars activates the grammar closest in color to the input (Figure 11).

**Figure 12:**
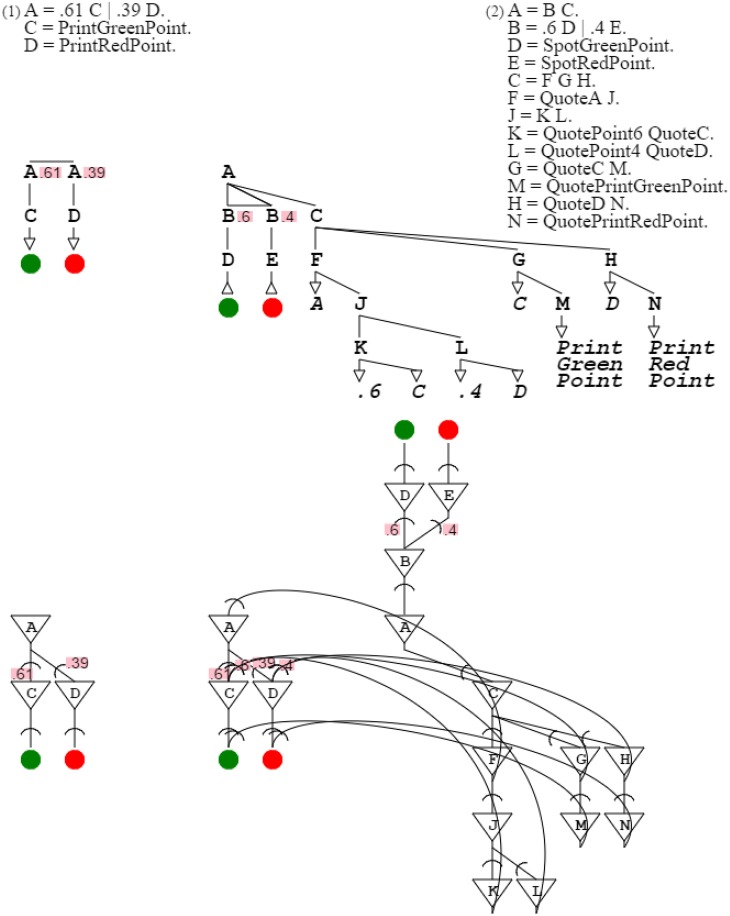
**Reinforcement learning**. The first grammar is the target orange grammar. The second grammar is the branch of the grammar swarm that is triggered when presented with a color close to orange. The first part of the second grammar is the triggering mechanism; the second part is the description of the target orange grammar. In the corresponding neural circuit, the top part is the trigger, the bottom right part is the description, and the bottom left part is the activated circuitry resolving to orange.

The swarm is then a tool of color detection. Let's assume that the swarm receives as input the color orange, that is, a mix of .61 green and .39 red. The branch of the swarm that detects that color is the second branch of the grammar since its probabilities, namely .6 and .4, are the closest to those of orange (the distribution of .6 and .4 is closest to that of .61 and .39). For that detection to be confirmed, this swarm branch needs to be connected to an orange production which acknowledges the detection and therefore presents an input for reinforcement. This capability is present once the swarm expands into production rules, as in “**A** = **B C**. **B** = .6 **D** | .4 **E**. **D** = *SpotGreenPoint*. **E** = *SpotRedPoint*. **C** = **F G H**. **F** = *QuoteA* **J**. **J** = **K L**. **K** = *QuotePoint6 QuoteC*. **L** = *QuotePoint4 QuoteD*. **G** = *QuoteC* **M**. **M** = *QuotePrintGreenPoint*. **H** = *QuoteD* **N**. **N** = *QuotePrintRedPoint*.” When the second branch is triggered, it allows closure of the reinforcement loop, which is validated by presentation of the expected orange color (Figure 12).

Any other gradient can be substituted to red-green, be it a different spectrum, tone, or other probabilistically-determined base for classification. However, this example of linking input to output for reinforcement learning needs to be augmented with more powerful capabilities. Consequently, I turn to demonstrating that the model is both general and organized.

### Self-description

I now show that probabilistic activation/recognition grammars can self-describe, allowing patterns to activate/recognize other patterns, such as with metaphor and composition.

**Figure 13:**
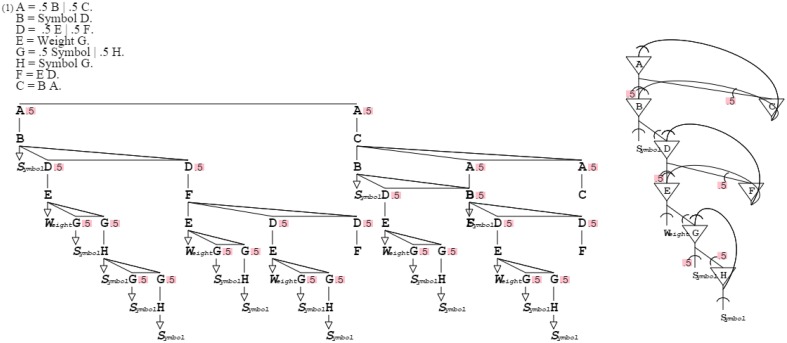
**The root grammar can describe any grammar, including itself**. Like the grammar it corresponds to, the neural circuit self-describes, providing root circuitry from which all circuits can derive.

Grammars-describing grammars can be recursively defined upward in an increasing generalization. The fixed point of that recursion is an ultimate grammar that can produce and recognize any grammar, including self. This root grammar “**A** = .5 **B** | .5 **C**. **B** = *Symbol* **D**. **D** = .5 **E** | .5 **F**. **E** = *Weight* **G**. **G** = .5 *Symbol* | .5 **H**. **H** = *Symbol* **G**. **F** = **E D**. **C** = **B A**.” defines the most general pattern, from which all other patterns derive (cf. Methods Summary, further below). Like all activation/recognition grammars, this top grammar can function in production, recognition, and mixed mode. The top grammar provides for a unified model since it covers all patterns (Figure 13).

This begs studying grammar learning over the full activation/recognition grammatical landscape. Kutzweil's two separate modules that I have not yet integrated in the theory are dealing with augmentation and organization. Augmentation is related to metaphor, the capability of creating new patterns as well as applying existing patterns to new domains. Organization is related to the general problem of associating patterns with one another. Metaphors allow reusing circuitry: for example, if a particular circuit counts items, one cannot imagine this circuit being replicated for the infinite number of possible items that can be counted. Therefore, there needs to be a mechanism that allows reusing the counting circuit to apply it to different items, which is what I will present. Organizational capabilities are an extension of metaphoric capabilities. If a circuit counts items, it should not only be capable to count any item, it should also be able to count anything; for example, the number of times an orange color is produced. In the following, I show that self-description allows handling both metaphor and organization within the Pattern Activation/Recognition Theory of Mind.

**Figure 14:**
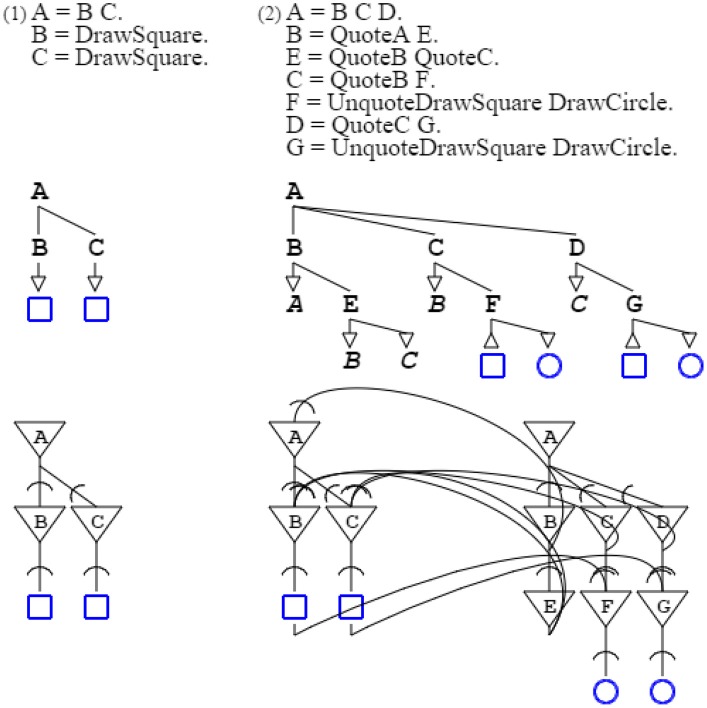
**Actualization of a metaphor**. The first grammar counts two squares, and is described by the second grammar. Each time the first grammar outputs a square, the second grammar transforms it into a circle, thereby allowing counting circles instead of squares. In the second neural circuit, the left part is the original square-counting circuit, the middle part activates the square-counting circuit, and the right part transforms counting squares into counting circles.

Regarding metaphors, a pattern can be described by another one, while the describing grammar can also perform other operations, a feature I have already used for swarms. For the sake of presentation, I consider a very simple pattern, that of counting two squares, done by grammar “**A** = **B C**. **B** = *DrawSquare*. **C** = *DrawSquare*.” This grammar is described by grammar “**A** = **B C D**. **B** = *QuoteA* **E**. **E** = *QuoteB QuoteC*. **C** = *QuoteB* **F**. **F** = *DrawSquare*. **D** = *QuoteC* **G**. **G** = *DrawSquare*.” This describing grammar can be augmented to draw a circle each time it detects a square, with “**A** = **B C D**. **B** = *QuoteA* **E**. **E** = *QuoteB QuoteC*. **C** = *QuoteB* **F**. **F** = *UnquoteDrawSquare DrawCircle*. **D** = *QuoteC* **G**. **G** = *UnquoteDrawSquare DrawCircle*.” The *unquote* operator is the reverse of the quote operator. With the addition of the new rules, instead of producing a square, the described grammar forwards the drawing operation to the describing grammar that then produces a circle. In other words, the describing grammar retargets the counting pattern from one domain (squares) to another (circles), which is the essence of metaphors, defined as “a cross-domain mapping in the conceptual system” (Lakoff, 1979). Of course, this is illustrating only the basic mechanism which metaphors rely on, while I have published elsewhere with Yi Mao a more complete account, using Montague grammars (Montague, 1974; du Castel and Mao, 2006) (Figure 14).

**Figure 15:**
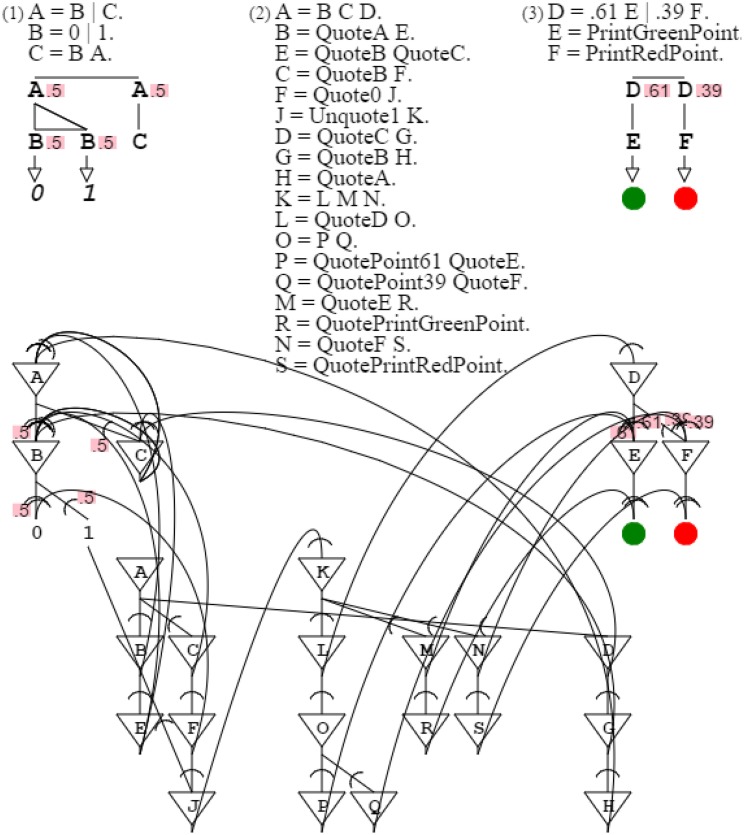
**Composition**. Grammars 1 and 3 are combined by grammar 2. The neural circuit of grammar 2 activates the neural circuit of grammar 1, and then the neural circuit of grammar 3, joining them to make them interdependent.

Regarding organization, I now consider two grammars previously discussed, digit grammar “**A** = **B** | **C**. **B** = *0* | *1*. **C** = **B A**.” and orange grammar “**A** = .61 **B** | .39 **C**. **B** = *PrintGreenPoint*. **C** = *PrintRedPoint*.” Digit grammar outputs digits, and orange grammar outputs points. Let's imagine that instead of producing the digit *1*, I want the digit grammar to produce an orange point, therefore producing a pattern of orange points that follows the pattern of *1*'s of the digit grammar. A way to do this could be to just replace digit *1* of the digit grammar by an orange grammar expansion, as in “**A** = **B** | **C**. **B** = *0* | **D**. **C** = **B A**. **D** = .61 **E** | .39 **F**. **E** = *PrintGreenPoint*. **F** = *PrintRedPoint*.” While this static combination produces the desired result, it does not provide a plausible model for the brain. Like in the case of metaphor discussed above, this process of combining grammars directly would accrue an infinity of combinations in the brain, not a possibility. What is needed is rather a means to combine the two circuits while preserving them, so that they can be combined in an infinite number of ways in a dynamic fashion. Here again, we turn to self-description, by invoking a grammar that describes these two grammars and combines them while keeping them intact. This is achieved by grammar “**A** = **B C D**. **B** = *QuoteA* **E**. **E** = *QuoteB QuoteC*. **C** = *QuoteB* **F**. **F** = *Quote0* **J**. **J** = *Unquote1* **K**. **D** = *QuoteC* **G**. **G** = *QuoteB* **H**. **H** = *QuoteA*. **K** = **L M N**. **L** = *QuoteD* **O**. **O** = **P Q**. **P** = *QuotePoint61 QuoteE*. **Q** = *QuotePoint39 QuoteF*. **M** = *QuoteE* **R**. **R** = *QuotePrintGreenPoint*. **N** = *QuoteF* **S**. **S** = *QuotePrintRedPoint*.” In addition to providing a means to reuse circuitry, this dynamic combination, by leaving the two circuits combined intact, allows them to be learned independently of each other. Independent learning helps limit the size of spaces explored by swarms (Bengio et al., 2009) (Figure 15).

While I have previously studied swarm learning for gradients, other variations can be considered, such as structural properties of grammars. In the same way as a grammar can generate a swarm of other grammars with different probabilities, it can generate other grammars with different structural properties, and then learn from the swarm, with branches that fulfill some target function. If learning the particular precedes the general, a possible but not necessary hypothesis for this argument, hierarchies of activation/recognition grammars are then learned from the bottom-up, accumulating experience in the memory of validated grammars until reaching the top grammar. In that model, pattern circuits build up over time, and the discovery of new patterns is an incremental operation.

### Methods summary

I now provide a method allowing better understanding of stochastic self-description, and I explain computation of the grammatical and neural diagrams.

Since grammar patterns are formal and structural, complex ones are better understood by considering them together with an expressive representation. For example, consider self-describing grammar “**A** = .5 **B** | .5 **C**. **B** = *Symbol* **D**. **D** = .5 **E** | .5 **F**. **E** = *Weight* **G**. **G** = .5 *Symbol* | .5 **H**. **H** = *Symbol* **G**. **F** = **E D**. **C** = **B A**.” For legibility, **A** can be recast as **Rules**, **B** as **Rule**, **C** as **RulesSequence**, **D** as **Alternates**, **E** as **Alternate**, **F** as **AlternatesSequence**, **G** as **Symbols**, and **H** as **SymbolsSequence**. Then the grammar is expressed as “**Rules** = .5 **Rule** | .5 **RulesSequence**. **Rule** = *Symbol* **Alternates**. **Alternates** = .5 **Alternate** | .5 **AlternatesSequence**. **Alternate** = *Weight* **Symbols**. **Symbols** = .5 *Symbol* | .5 **SymbolsSequence**. **SymbolsSequence** = *Symbol* **Symbols**. **AlternatesSequence** = **Alternate Alternates**. **RulesSequence** = **Rule Rules**.” This method can be generalized to any grammar for better understanding of their function.

Earlier versions of some of the grammars were posted online as unpublished works and are presented here in new versions with permission from the author (du Castel, 2013a,b).

**Figure 16:**
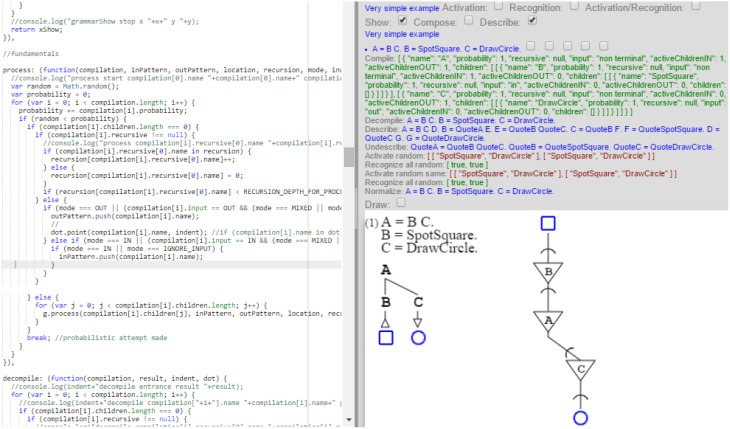
**This is the implementation**. The left side shows executing code, while the right side shows the result of execution for a simple grammatical and neural description.

The model is fully implemented as a prototype which I ran to produce the grammatical and neural diagrams of this article as well as all describing grammars. The prototype compiles grammars expressed in Wirth's format into both their graphical expression and the expression of their self-description. These compilations drive execution of the stochastic grammars in two modes: one provides the grammatical diagrams, while the other provides the neural diagrams. This unity of execution ensures that both diagrams are indeed issued from a single description, as presented by the model, and that there is in fact a one-to-one correspondence between the grammars and their neural representations. The prototype is quite elaborate in its treatment of grammars, as it handles all the cases of this article and more, but is less so in its handling of sampling and distributions, where it is far from the state of the art. While the prototype implementation is not in a form appropriate for general use, I present next a screen shot showing both the core execution code and the results of the execution on a very simple example (Figure 16).

My implementation of self-describing activation/recognition stochastic grammars, which affords universal computation, meta-circularity, and probabilities, belongs, however modestly, to a general class of languages well-represented by PRISM (Sato and Kameya, 1997), which is a probabilistic offspring of Prolog, and Church (Goodman et al., 2008), which is a probabilistic offspring of Lisp. From a theoretical perspective, a difference between the three is that PRISM is oriented toward logic, Church toward general modeling, while the present implementation is oriented toward properties discussed here: activation/recognition symmetry, grammar/neural diagrams, grammatical inference, metaphors, and composition. Being universal, the three languages support each other's computational model, but in each case less naturally than with the original. To further illustrate this point, a language like Mathematica (Wolfram, 1988) presents similar capabilities but is rather a natural fit to mathematics at large. Perhaps anecdotally, I suggest that the less natural character of advanced logic, probabilistic modeling, or sophisticated mathematics transcribed by self-describing activation/recognition stochastic grammars reflects well the reality of human performance, where each of these activities involves pragmatic graduate-level experience, whereas grammars are arguably inherently a construct of early childhood and a more natural human experience. This said, from a practical perspective, my prototype could be implemented as a new module of PRISM and Church, focused on the self-describing activation/recognition grammatical and neural functions of this article but benefiting from the statistical capabilities and support found in these two systems.

## Results

While grammars are one of the oldest subjects of scientific study, neither activation/recognition grammars nor the theoretical model of stochastic grammars recursively describing other grammars hierarchies have, to my knowledge, been studied before. This model describes circuits in stochastic relationships; furthermore, it describes that very description, and keeps doing so recursively up to self-description. At each recursion patterns can be produced, recognized, and learned via reinforcement and composition, building up from past descriptions.

Following is a recapitulation of the figures in this article showing grammars, their schema, and their neural circuit, with comments now framed by the whole of this article:

**Table:** 

Figure 1:	Activation/recognition grammars correspond to neural circuits, with recognition represented by cell input, and activation by cell output.
Figure 2:	Activation/recognition grammars express both parallel and sequential execution in neuronal circuits.
Figure 3:	Circuits of activation/recognition grammars represent concepts in both space and time.
Figure 4:	Probabilities of activation/recognition grammars map synaptic probabilities.
Figure 5:	Recursion in activation/recognition grammars corresponds to recurrence in neural circuits, via autapses or regular synapses.
Figure 6:	An activation/recognition grammar and its neural circuit can represent a Turing machine, asserting universal computation.
Figure 7:	Activation/recognition grammars with varying terminals express a metaphor in neural circuits.
Figure 8:	By varying probabilities, activation/recognition grammars and their circuits can express gradient patterns.
Figure 9:	An activation/recognition grammar can describe another one, and its neural circuit activates the circuit of the grammar described.
Figure 10:	Activation of one circuit by another projects probabilities from the latter to the former.
Figure 11:	An activation/recognition grammar can express a swarm of similar circuits.
Figure 12:	A branch of a swarm of neural circuits enables reinforcement learning by forwarding results to another circuit.
Figure 13:	The root grammar can describe any other grammar, including self, and *ditto* for the corresponding root neural circuit.
Figure 14:	A self-describing neural circuit activates a metaphor by directing the output of another neural circuit.
Figure 15:	Any two circuits can be connected dynamically by a third one using self-description.

With this general exhibit of properties of activation/recognition grammars and their neural circuits, a model emerges that allows neural computations to be expressed in the well-researched mathematical framework of grammar theory. With their corresponding neural circuits, self-describing activation/recognition grammars form a biologically-inspired model that can produce, recognize, and learn neurons and circuits of neurons from a single source, suggesting a unitary model for their development and function.

## Discussion

### Theory

My goal was to provide a unified model that augments and completes Kurzweil's theory. Activation/recognition grammars further that theory by allowing both activation and recognition of patterns; this corresponds to neural activation and recognition functions, including motor and sensory stimuli.

Stochastic grammars are one step up from Kurzweil's HHMMs, allowing full recursion, which maps naturally with neural circuits that are analogically recurrent (Gilbert and Li, 2013). The combination of activation/recognition with probabilities warrants adaptive pattern processing that exploits the stochastic nature of synaptic connections (Staras and Branco, 2009).

By using activation/recognition stochastic grammars with swarming patterns, it is possible to include learning in the model. By combining self-description with these properties, learning extends from simple patterns to patterns of patterns, allowing the treatment of consistency checking as yet another pattern.

Stochastic grammars can both describe patterns and generate new ones, using a combinatorial learning process that allows varying patterns for purpose. New patterns are modifications of existing ones, which expresses Kurzweil's metaphoric model of discovery.

### Validation

I now discuss in regards to validation three claims of the new theory, which are: patterns are governing throughout; activation and recognition share descriptions; and patterns are self-describing. In terms of neural computation, the algebra is that of stochastic grammars; neural circuits implement their rules; and computation stems from recursively nested descriptions.

Back-propagation (LeCun et al., 1998) and spiking (Maass, 1997) networks are perhaps paragons of current computational artificial neural networks. The former provides strong results on recognition benchmarks while aiming at biological plausibility (Bengio et al., 2015); the latter roots more in biology while aiming at matching the former in performance (Schmidhuber, 2015). Neither addresses acquisition/recognition symmetry nor self-description.

I suggest, however, that acquisition/recognition symmetry and self-description are not in conflict with current artificial networks, but are rather complimentary to them, so that the way forward may be a mix of current practice blended with the new requirements. Another possibility is that a new brand of analytical (Carpenter and Grossberg, 1987; Herbort and Butz, 2012) neural networks based on stochastic grammars and their distributional and sampling properties emerges with millions of diachronically and synchronically learned patterns (Rodríguez-Sánchez et al., 2015). Additionally, the same principles can be applied to structuring biological artificial neural networks (Markram, 2012).

The symmetry of activation and recognition has been theorized in particular with the introduction of common coding (Prinz, 1990), and of a shared circuit model (Hurley, 2007) supported by the discovery of mirror neurons (Gallese et al., 1996). The new theory supports activation and recognition sharing a same circuit as well as them sharing a same description but with different circuitry. It can therefore be proposed as an underlying formalism for both common coding (same description) and the shared circuit model (same circuit). Neurophysiological evidence for these models would then favor the new theory as well. Nevertheless, I think that definite validation entails mapping the circuitry presented in this paper (Mikula and Denk, 2015).

While stochastic grammatical self-description is new, it finds a home in theories of neural reuse (Anderson, 2010) and cultural recycling (Dehaene and Cohen, 2007). In both theories, it has been recognized that co-optation of an existing circuit for a new function does not negate pre-existing functions. For example, hand gestures accompanying speech reflect the source domain of a spoken metaphor (McNeill, 1992; Marghetis et al., 2014). This is in accord with the activation mechanism of this article, which only adds, not substitutes, to the output of a neuron. New and old functions coexist, and may or not find an outward expression, depending on activation of downward circuitry. When it does, behavior consistent with the theory ensues, such as gestures associated with speech. General validation, however, would consist in a broad understanding of conditions under which situations of co-opted neural output actually affect behavior.

With the above examples a comprehensive underpinning model of neural circuitry emerges, but ultimate validation of the theory relates to the unity of the brain. Indeed, the theory suggests that neural circuits are composed by self-description into circuitry of increasing generality, up to the root stochastic grammar describing all stochastic grammars. Would neuroscience reach the point where neural circuits can be followed along that path (Laumonnerie et al., 2015), direct validation of the theory would then be effected, arguably advancing a formal version of Aristotle's hylomorphism.

### Conclusion

I must emphasize that the Pattern Activation/Recognition Theory of Mind should reflect more than the neuronal circuitry that I have considered. In particular, neuron/interneuron distinctions (DeFelipe et al., 2013), columns and layers arrangements, modular specialization, and brain waves, are to be part of an elaboration of the model. While I do not know that any or more of these properties would invalidate the model at this point, I would, *a contrario*, like them to reinforce it.

But crucially, the model predicts that we should find in the brain self-descriptive neural circuits of the kind modeled by the theory. That would be the discovery that shows that this new model of the brain answers the millenary quest for understanding the unity of mind.

### Conflict of interest statement

The author declares that the research was conducted in the absence of any commercial or financial relationships that could be construed as a potential conflict of interest.
